# Draft Genome Sequence of *Pseudomonas frederiksbergensis* SI8, a Psychrotrophic Aromatic-Degrading Bacterium

**DOI:** 10.1128/genomeA.00811-15

**Published:** 2015-07-16

**Authors:** Oscar N. Ruiz, Lisa M. Brown, Richard C. Striebich, Susan S. Mueller, Thusitha S. Gunasekera

**Affiliations:** a Air Force Research Laboratory, Aerospace Systems Directorate, Fuels and Energy Branch, Wright-Patterson AFB, Ohio, USA; b University of Dayton Research Institute, Dayton, Ohio, USA

## Abstract

*Pseudomonas frederiksbergensis* strain SI8 is a psychrotrophic bacterium capable of efficient aerobic degradation of aromatic hydrocarbons. The draft genome of *P. frederiksbergensis* SI8 is 6.57 Mb in size, with 5,904 coding sequences and 60.5% G+C content. The isopropylbenzene (cumene) degradation pathway is predicted to be present in *P. frederiksbergensis* SI8.

## GENOME ANNOUNCEMENT

*Pseudomonas frederiksbergensis* strain SI8 was isolated from a desert soil sample from under a fuel storage bladder in Kuwait*.* This strain grows in temperatures ranging from 4°C to 30°C and metabolizes toluene, ethylbenzene, and propylbenzene, among other compounds. Based on the 16S rRNA gene sequence, *P. frederiksbergensis* SI8 is at least 99.0% similar to *P. frederiksbergensis* strains V1R0E2, JW-SD2, DSM13022, CB2B1. An alkane-degrading psychrophilic *P. frederiksbergensis* strain and a psychrotrophic strain from soil at a coal gasification plant have been reported (1, 2). Due to the importance of cold-tolerant microorganisms in the environment and their potential application in bioremediation, we have sequenced the genome of *P. frederiksbergensis* SI8, to understand the underlying aromatic-degradation mechanisms.

*P. frederiksbergensis* SI8 was sequenced on a Roche 454-GS Junior platform using a whole-genome shotgun (WGS) approach, resulting in 728,652 reads. The sequence reads were assembled with the Roche *de novo* Assembly software. More than 99% or 724,816 reads were assembled into 65 large (>500 bp) contigs, with an *N*_50_ of 320,712 bp. The longest contig was 684,101 bp. The draft genome sequence was 6,569,267 bases in length, with a G+C content of 60.5%. Rapid genome annotation using RAST annotation server (3) described 5,904 coding sequences (CDSs) and 64 RNAs. The coding sequences were classified into 546 subsystems of which amino acids and derivatives (*n* = 671 CDSs); carbohydrates (*n* = 509); membrane transport (*n* = 282); protein metabolism (*n* = 235); fatty acids, lipids, and isoprenoids (*n* = 222); RNA metabolism (*n* = 210); stress response (*n* = 206); cell wall and capsule (*n* = 186); virulence, disease, and defense (*n* = 159); respiration (*n* = 155); metabolism of aromatic compounds (*n* = 146); motility and chemotaxis (*n* = 143); regulation and cell signaling (*n* = 127); and DNA metabolism (*n* = 144) were the most abundant.

The NCBI Prokaryotic Genome Annotation Pipeline (http://www.ncbi.nlm.nih.gov/genome/annotation_prok/) predicted the benzene 1,2-dioxygenase, vanillate monooxygenase, catechol 1,2-dioxygenase (*catA*), homogentisate 1,2-dioxygenase, and 4-hydroxybenzoate 3-monooxygenase genes. The *cum* gene cluster (4) for isopropylbenze (cumene) degradation was present. This cluster is 99% similar to that of *P. fluorescens*, accession number D37828. *P. frederiksbergensis* SI8 showed at least 71% homology with twenty of the twenty one genes, with the exception of *ditE*, of the *dit* cluster for abietane diterpenoids catabolism of *P. abietaniphila*, accession number AF119621 (5). Additionally, the genes encoding protocatechuate 3,4-dioxygenase (*ligA* and *ligB*), 3-carboxymuconate cycloisomerase, and 4-carboxymuconolactone decarboxylase, of the central protocatechuate catabolic pathway for aromatic degradation were observed (6). The gentisate 1,2-dioxygenase (*gdo*) gene essential for the gentisate catabolic pathway of polycyclic aromatics was not detected (6). Important hydrocarbon degradation genes encoding 2,4-dichlorophenol 6-monooxygenase, alkanesulfonate monooxygenase, 2-polyprenyl-6-methoxyphenol hydroxylase, aminobenzoate oxygenase, and 2-nitropropane dioxygenase were observed. A gene cluster with at least 80% homology to the *ttg*2 operon encoding an ABC-type transporter essential for toluene resistance in *P. Putida* and *P. aeruginosa*, was observed (7, 8). The *alkB* genes were not detected, explaining its inability to degrade *n*-alkanes. This genome will provide valuable insight into the genetics and metabolism of hydrocarbon-degrading psychrotrophic bacteria.

### Nucleotide sequence accession number. 

This project has been deposited in DDBJ/EMBL/GenBank under the accession number JQGJ00000000.
